# Supplementary figures and images for: HIV, sexual violence, and termination of pregnancy among adolescent and adult female sex workers in Malawi: A respondent-driven sampling study
Source: PLoS One. 2022 Dec 30;17(12):e0279692. doi: 10.1371/journal.pone.0279692 (PMC9803093; doi:10.1371/journal.pone.0279692)

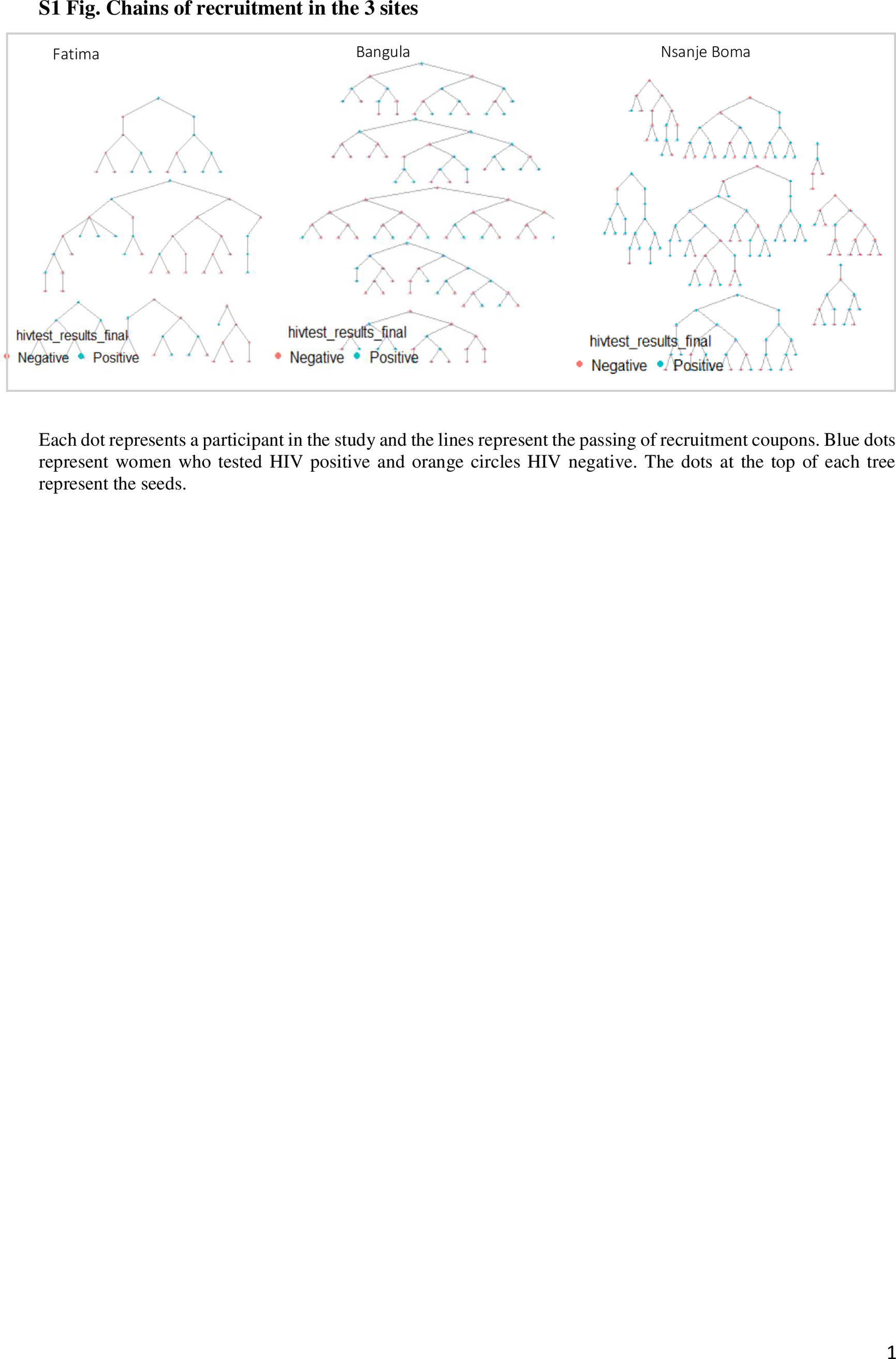

Supplement: S1 Fig — Each dot represents a participant in the study and the lines represent the passing of recruitment coupons. Blue dots represent women who tested HIV positive and orange circles HIV negative. The dots at the top of each tree represent the seeds. (TIF) [file pone.0279692.s001.tif]
