# Supplementary material for: HIV, sexual violence, and termination of pregnancy among adolescent and adult female sex workers in Malawi: A respondent-driven sampling study
Source: PLoS One. 2022 Dec 30;17(12):e0279692. doi: 10.1371/journal.pone.0279692 (PMC9803093; doi:10.1371/journal.pone.0279692)
Supplement: S2 Fig — a/HIV prevalence; b/Age group; c/Ever enrolled in MSF activities. (PDF) [file pone.0279692.s002.pdf]

S2 Fig. Diagnosis analysis

a/HIV prevalence

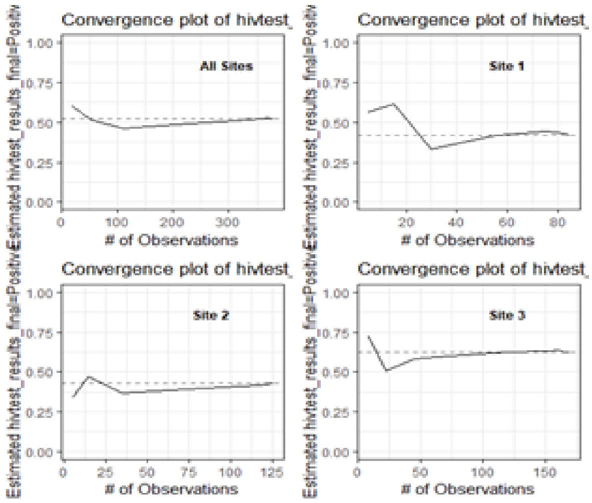

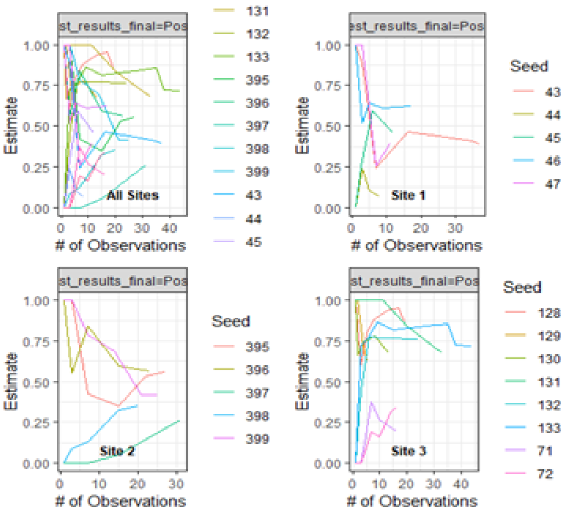

Figure 2A *Figure 2B*

HIV prevalence estimates over waves of recruitment, overall and by sites (Figure 2A), and for each seed (Figure 2B). Site 1: Fatima, Site 2: Bangula, Site 3: Nsanje Boma

Convergence plots: The overall RDS-II HIV prevalence estimates appeared to converge (Figure 2A). At the start of recruitment, the prevalence estimate was 60%, which dropped to 45% around the 100th recruit (one-third of the overall sample) and then steadily approached the final estimate of 52% (indicated by the dotted line) at the end of recruitment. However, estimate equilibrium was not yet reached as the line showed a steady upward trend and no sign of flattening out. This was also true for site 2, but not for site 1 and site 3. Here, the lines flattened out midway (site 1) or two-thirds of the way (site 3) through recruitment process indicating equilibrium reached and the estimate has stabilised.

Bottleneck plots: Bottleneck plots of the RDS-II HIV prevalence estimates showed important changes in estimates over the course of recruitment (Figure 2B). In general, recruitment chains whose prevalence estimates started at 100% dropped steeply as recruitment progressed and HIV negatives were enrolled and vice versa for recruitment chains starting with 0% prevalence. However, not all chains converged towards the overall RDS-II population estimate of 52% and several bottlenecks were visible, especially several chains stabilising at higher prevalence estimates due to site variation.

b/Age group

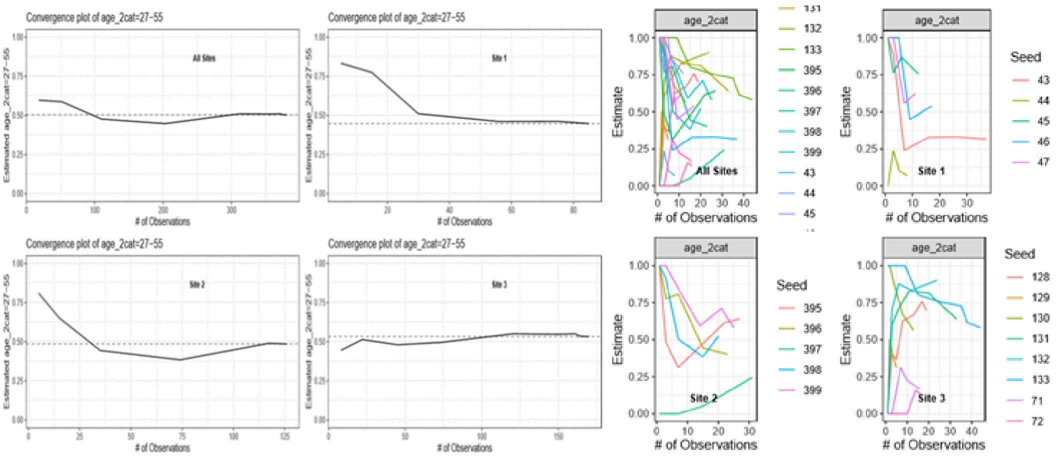

Figure 3A *Figure 3B*

Age group estimates over waves of recruitment, overall and by sites (Figure 3A), and for each seed (Figure 3B). Site 1: Fatima, Site 2: Bangula, Site 3: Nsanje Boma

Convergence plots: Convergence analysis was performed obtaining the RDS-II estimates for the proportion of FSW aged 27-55 years. Estimate equilibrium was attained relatively early (Figure 3A). At the start, the estimate was 60%, and as the number of participants increased, the estimate dropped to 45% and then increased to 50% at the end of recruitment. Both site 1 and 2 recruited older FSW at the start, hence estimate was close to 80% but then dropped quickly and stabilised to 50% as younger FSWs were recruited. Site 3 recruited a more diverse age range since the start, as initial estimate of clients aged 27-55 years was 40% which steadily increased and stabilised at 50%.

Bottleneck plots: The bottleneck plots revealed recruitment chains in site 1 and site 2 starting with older FSW but recruitment quickly crossed over to the younger age groups as lines descended steeply from 100% (Figure 3B). In site 3, there was cross-over in both directions from older to younger and younger to older. Across all sites, there were a couple of chains that remained caught within the younger age group (1 in site 1, 1 in site 2 and 2 in site 3).

c/Ever enrolled in MSF activities

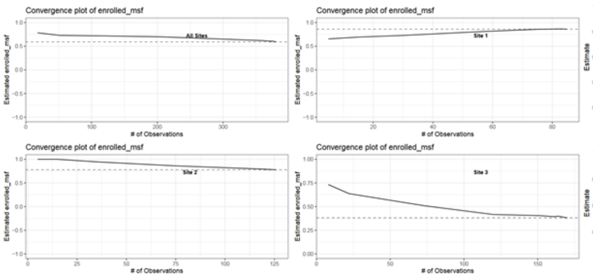

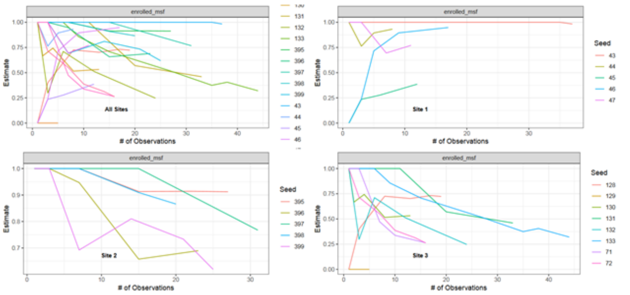

Figure 4A *Figure 4B*

Ever enrolled in MSF estimates over waves of recruitment, overall and by sites (Figure 4A), and for each seed (Figure 4B). Site 1: Fatima, Site 2: Bangula, Site 3: Nsanje Boma

Convergence plots: Convergence analysis was also performed obtaining the RDS-II estimates for the proportion of FSWs who were ever enrolled in MSF activities (Figure 4A). At the start, the estimate was close to 75%, and as the number of participants increased, the estimate dropped to 50%. Both site 1 and 2 recruited more FSWs who were ever enrolled in MSF activities since the start (in site 2, all seeds were previously enrolled with MSF). Site 3 recruited a more diverse group of FSWs in term of ever-enrolment with MSF since the beginning, and the estimate decreased more than in the two other sites suggesting more FSWs outside of MSF network were recruited until the estimate stabilized around 35%.

Bottleneck plots: Site 1 was dominated by 1 chain in particular which recruited up-to 30 people, all ever enrolled in MSF activities, whereas the other 4 chains were relatively short (Figure 4B). In the 2 others sites, the recruitment chains were longer. In site 2 and 3, the majority of initial seeds (all in site 2) were ever enrolled in MSF activities and then more FSWs outside of MSF network were recruited.
