# Supplementary material for: HIV, sexual violence, and termination of pregnancy among adolescent and adult female sex workers in Malawi: A respondent-driven sampling study
Source: PLoS One. 2022 Dec 30;17(12):e0279692. doi: 10.1371/journal.pone.0279692 (PMC9803093; doi:10.1371/journal.pone.0279692)
Supplement: Attachment — Submitted filename: Minor comments.docx [file pone.0279692.s003.docx]

**HIV, sexual violence, and termination of pregnancy among adolescent and adult female sex workers in Malawi: a respondent-driven sampling study**

The cross-sectional bio-behavioural survey was conducted among younger FSWs in 3 purposively selected sites out of 5 existing intervention areas (surrounding predominantly male worksites and transport routes). The researcher used the RDS method to reach the hard-to-reach populations. Trained counsellors were collected the data in prior identified venues to ensure confidentiality from consented participants.

I congratulate the researcher/team for the effort to implement the RDS method in Malawi as the first kind of nature; this method may enable the researcher to capture intervention and non-intervention FSWs in the study, to some extent.

Minor comments

1. Giving information about urban and rural variations observed in HIV prevalence and other sexual behaviours indicators will be helpful for programme implementation by The Malawi National AIDS Commission, Malawi.
2. In general, the 15-24 age group HIV positive case is considered as a proxy for incidence since the study focussed only on younger FSWs. Removing the known HIV positives from databases and calculating the incidence % may be helpful to the programme.
3. In table 2, row (locations for providing SW) under the Nsanje Mboma site, N needs to be replaced by the actual number.
